# Expression of multidrug resistance-associated protein1,P-glycoprotein, and thymidylate synthase in gastric cancer patients treated with 5-fluorouracil and doxorubicin-based adjuvant chemotherapy after curative resection

**DOI:** 10.1038/sj.bjc.6600305

**Published:** 2002-05-03

**Authors:** J-H Choi, H-Y Lim, H J Joo, H S Kim, J W Yi, H C Kim, Y K Cho, M W Kim, K B Lee

**Affiliations:** Department of Hematology-Oncology, Ajou University School of Medicine, Suwon, 442-721, Korea (Rep.); Department of Pathology, Ajou University School of Medicine, Suwon, 442-721, Korea (Rep.); Department of Surgery, Ajou University School of Medicine, Suwon, 442-721, Korea (Rep.)

**Keywords:** gastric cancer, multidrug resistance-associated protein1, P-glycoprotein, thymidylate synthase, adjuvant chemotherapy, prognosis

## Abstract

Both 5-fluorouracil and doxorubicin are commonly used agents in chemotherapy of gastric cancer in adjuvant setting as well as metastatic disease. In a variety of malignancies, high expression of multidrug resistance-associated protein1 and P-glycoprotein has been associated with resistance to doxorubicin, whereas 5-fluorouracil resistance has correlated with the level of thymidylate synthase expression. We evaluated the expression of multidrug resistance-associated protein1, P-glycoprotein, and thymidylate synthase using immunohistochemistry in 103 locally advanced gastric cancer patients (stage IB-IV) who underwent 5-fluorouracil and doxorubicin-based adjuvant chemotherapy after curative resection and investigated the association between their expression and clinicopathologic characteristics including prognosis of the patients. While high expression (⩾5% of tumour cells positive) of multidrug resistance-associated protein1 and P-glycoprotein was observed in 70 patients (68%) and 42 patients (41%), respectively, 65 patients (63%) had primary tumours with high expression (⩾25% of tumour cells positive) of thymidylate synthase. There was a significant association between multidrug resistance-associated protein1 and P-glycoprotein expression (*P*<0.0001) as well as P-glycoprotein and thymidylate synthase expression (*P*<0.0001). High multidrug resistance-associated protein1 and P-glycoprotein expressions were associated with well and moderately differentiated histology (*P*<0.0001 and *P*=0.03, respectively) and intestinal type (*P*<0.0001 and *P*=0.009, respectively). High multidrug resistance-associated protein1 expression correlated with lymph node metastasis (*P*=0.037), advanced stage (*P*=0.015), and older age (*P*=0.021). Five-year disease-free survival and overall survival of total patients were 55.2% and 56.2%, respectively, with a median follow-up of 68 months. There were no significant differences in disease-free survival and overall survival according to the expression of multidrug resistance-associated protein1 (*P*=0.902 and *P*=0.975, respectively), P-glycoprotein (*P*=0.987 and *P*=0.955, respectively), and thymidylate synthase (*P*=0.604 and *P*=0.802, respectively). Concurrent high expression of these proteins (high multidrug resistance-associated protein1/P-glycoprotein, high multidrug resistance-associated protein1/thymidylate synthase, high P-glycoprotein/thymidylate synthase) did not correlate with disease-free survival or overall survival. Even high expression of all three proteins was not associated with poor disease-free survival (*P*=0.919) and overall survival (*P*=0.852). In conclusion, high expression of multidrug resistance-associated protein1, P-glycoprotein, and thymidylate synthase did not predict poor prognosis of gastric cancer patients treated with 5-fluorouracil and doxorubicin-based adjuvant chemotherapy. A larger study including patients treated with surgical resection alone would be necessary.

*British Journal of Cancer* (2002) **86**, 1578–1585. DOI: 10.1038/sj/bjc/6600305 www.bjcancer.com

© 2002 Cancer Research UK

## 

Gastric cancer is the most common malignancy in many countries including Korea (Fuchs and Mayer, 1995; Suh *et al*, 2000). The prognosis of patients with locally advanced gastric cancer is generally poor even with curative resection, which is an essential treatment for the long-term survival of patients (Noguchi *et al*, 1989; Fuchs and Mayer, 1995). Adjuvant chemotherapy has failed to demonstrate a significant survival benefit for such patients in most randomized trials and meta-analyses although a recent study showed improved survival with postoperative chemoradiotherapy compared to surgery alone (Krook *et al*, 1991; Hermans *et al*, 1993; Fuchs and Mayer, 1995; Lise *et al*, 1995; Macdonald *et al*, 1995; Kelsen, 1996; Kim *et al*, 1998; Cirera *et al*, 1999; Earle and Maroun, 1999; Macdonald *et al*, 2001).

In adjuvant chemotherapy of gastric cancer, 5-fluorouracil (5-FU) and doxorubicin have been commonly used in combination (Krook *et al*, 1991; Hermans *et al*, 1993; Lise *et al*, 1995; Macdonald *et al*, 1995; Kelsen, 1996; Kim *et al*, 1998; Earle and Maroun, 1999). Since the intrinsic or acquired resistance of cancer cells to these agents is the most important cause of the failure of adjuvant chemotherapy, identification of molecules associated with the resistance of tumours to anticancer drugs can provide valuable information in adjuvant chemotherapy of gastric cancer.

In terms of resistance to 5-FU, thymidylate synthase (TS), which is a critical target of 5-FU, has been widely investigated. TS catalyses the methylation of deoxyuridine monophosphate to deoxythymidine monophosphate, which is an essential process for DNA synthesis (Pinedo and Peters, 1988). High expression of TS may be associated with 5-FU resistance in a variety of malignancies including gastric cancer (Johnston *et al*, 1992, 1995; Lenz *et al*, 1996). For doxorubicin, multidrug resistance (MDR), which means the resistance of cancer cells against several anticancer drugs, has been considered as one of the important causes of drug resistance (Goldstein *et al*, 1989; Gottesman and Pastan, 1993). The best-characterised mechanism of MDR is overexpression of P-glycoprotein (P-gp) encoded by MDR1 gene (Goldstein *et al*, 1989; Gottesman and Pastan, 1993). P-gp is 170-Kda transmembrane protein and functions as an adenosine triphosphate-dependent drug efflux pump (Goldstein *et al*, 1989; Gottesman and Pastan, 1993). It is responsible for resistance of cancer cells to a wide range of structurally unrelated cytotoxic agents including doxorubicin, etoposide, vinca alkaloid, and actinomycin-D (Goldstein *et al*, 1989; Gottesman and Pastan, 1993). In addition to P-gp, multidrug resistance-associated protein1 (MRP1), 190-Kda membrane bound glycoprotein encoded by MRP1 gene, has been found to be involved in the resistance of cancer cells to the same category of anticancer drugs as in P-gp with similar mechanism (Cole *et al*, 1992; Hipfner *et al*, 1999). Moreover, MRP1 expression has been associated with resistance to chemotherapeutic agents or poor survival in breast cancer, neuroblastoma, lung cancer, and gastric cancer (Endo *et al*, 1996a,b; Norris *et al*, 1996; Nooter *et al*, 1997; Young *et al*, 1999).

In gastric cancer patients who underwent surgical resection with or without adjuvant chemotherapy, several investigators have reported the prognostic significance of TS, MRP1, and P-gp with conflicting results (Endo *et al*, 1996a; Monden *et al*, 1997; Kuniyasu *et al*, 1998; Takebayashi *et al*; 1998; Suda *et al*, 1999; Choi *et al*, 2001). We have recently reported that expression of TS did not predict poor survival in 103 gastric cancer patients treated with 5-FU and doxorubicin-based adjuvant chemotherapy after curative resection (Choi *et al*, 2001). We evaluated the expression of MRP1, P-gp, and TS using the same cohort with longer follow-up and investigated the association between their expression and various clinicopathologic characteristics including prognosis of the patients.

## MATERIALS AND METHODS

### Patients

Included in this study were 103 patients with locally advanced gastric adenocarcinoma who underwent 5-FU and doxorubicin-based adjuvant chemotherapy after curative surgical resection at Ajou University Medical Center in Suwon, Korea, between July 1994 and October 1996. All patients had a postsurgical pathologic stage ranging from IB to IV without evidence of distant metastasis according to the American Joint Committee on Cancer (1997) TNM classification. Patients with distant abdominal lymph node metastasis (M1) were excluded.

In terms of adjuvant chemotherapy, the most commonly administered regimen was FA (5-FU, doxorubicin) with OK-432 (51 patients), followed by FAM (5-FU, doxorubicin, mitomycin-C) (33 patients), FA (11 patients), and FA with lentinan (eight patients). Chemotherapy was usually started 2–3 weeks after surgery. In FA regimen with or without immunotherapy (OK-432 or lentinan), 5-FU (500 mg m^−2^ per day) was given by intravenous (i.v.) infusion for 30 min on days 1, 8 and 15, and doxorubicin (40 mg m^−2^) was given by rapid i.v. injection on day 1. The treatment was repeated every 3 weeks for 12 cycles. OK-432 (2.0 Klinishe Einheit/day) and lentinan (2 mg/day) were administered intramuscularly and intravenously, respectively, weekly throughout the FA chemotherapy period. In FAM regimen, 5-FU (1000 mg m^−2^ per day) was given by continuous i.v. infusion on days 1 to 3, and doxorubicin (40 mg m^−2^) and mitomycin-C (10 mg m^−2^, every other cycle) were administered by rapid i.v. injection on day 1. The treatment was repeated every 4 weeks for 12 cycles and mitomycin-C was administered every 8 weeks. The other characteristics of patients including the definition of curative section and follow-up schedule after treatment were previously described (Choi *et al*, 2001).

### Immunohistochemical staining

Immunohistochemical staining of formalin-fixed, paraffin-embedded tumour tissue was performed using MRPr1 monoclonal antibody against MRP1 (Signet Laboratories, Dedham, MA, USA), JSB-1 monoclonal antibody against P-gp (Signet Laboratories, Dedham, MA, USA), and TS 106 monoclonal antibody against TS (Neomarkers, Fremont, CA, USA) as described previously (Choi *et al*, 2001).

Formalin-fixed, paraffin-embedded sections of colon adenocarcinoma known to have high expression of TS and normal colon tissue were used as positive controls for TS and P-gp, respectively. The negative controls for TS and P-gp were made by the omission of the primary antibody during the process of immunohistochemical staining. For MRP1, cytospin preparation of the MRP1-overexpressing doxorubicin-resistant human lung cancer cell line GLC4/ADR and its drug-sensitive parental cell line GLC4, which were kindly provided by Dr EG de Vries (Groningen University, Groningen, The Netherlands), were used as positive and negative control, respectively.

### Tissue evaluation

The slides were examined independently by two observers (H.J.J. and K.B.L) blinded to both clinical and pathologic data. The expression of TS was divided into high TS (greater than or equal to 25% of tumour cells positive) and low TS (less than 25% of tumour cells positive or no staining) groups as reported previously (Choi *et al*, 2001). The expression of MRP1 and P-gp was also classified into high (greater than or equal to 5% of tumour cells positive) and low (less than 5% of tumour cells positive or no staining) based on the extent of staining.

### Statistical analysis

Disease-free survival (DFS) and overall survival (OS) were calculated using Kaplan–Meier Method (Kaplan and Meier, 1958). Disease-free survival was defined as the time from the day of operation to a documented recurrence, or second primary cancer, or death from any other cause. Data on patients who did not have a recurrence were censored at the last follow-up. Overall survival was defined as the time from the day of operation to death; data on survivors were censored at the last follow-up. The differences between the survival curves were tested by using the log-rank test. Comparison of variables according to the expression of drug-resistance proteins was evaluated with the Student's *t*-test and chi-square test.

## RESULTS

### Patient characteristics

Of 103 patients who were assessable to drug resistance proteins, 71 were male and 32 were female, and their median age was 53 years (range, 28 to 72). Stages were IB in 9, II in 28, IIIA in 31, IIIB in 17, and IV in 18 patients. The median number of chemotherapy cycles was 8 (range, 1 to 13).

### Association of expression of drug resistance proteins with patient and tumour characteristics

TS staining pattern in gastric tumours and typical examples were previously reported (Choi *et al*, 2001). Within the study group, 65 patients (63%) had primary tumours with high TS expression (⩾25% of tumor cells positive), and 38 patients (37%) demonstrated low TS expression (<25% of tumour cells positive or no staining). The relationship between TS expression and patient and tumour characteristics was also previously reported (Choi *et al*, 2001). High TS expression was associated with poorly differentiated histology (*P*=0.015) and mixed type tumours in Lauren's classification (*P*=0.027).

**Figure 1:**
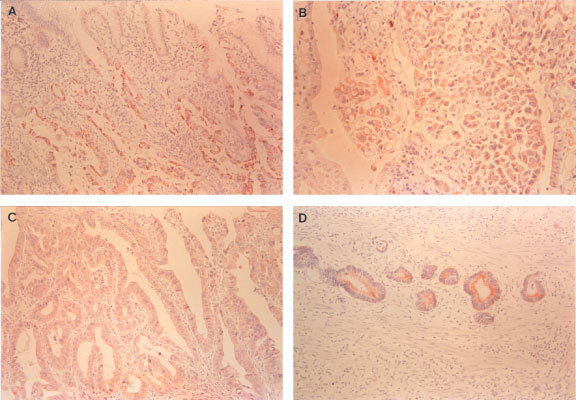
Immunohistochemical staining of MRP1 and P-gp in gastric cancer. (**A**) Tubular adenocarcinoma showing strong immunoreactivity for MRP1 compared to normal foveolar epithelium (×100). (**B**) Signet ring cell carcinoma showing strong cytoplasmic staining of MRP1 compared to non-neoplastic epithelium (×200). (**C**) Tubulopapillary adenocarcinoma showing diffuse immunoreactivity for P-gp (×200). (**D**) Strong expression of P-gp in cytoplasm of tubular adenocarcinoma (×200).

Both MRP1 and P-gp staining pattern in gastric tumours showed predominantly cytoplasmic staining in tumour cells (Figure 1

). High expression (⩾5% of tumour cells positive) of MRP1 and P-gp in primary tumour was observed in 70 patients (68%) and 42 patients (41%), respectively. High MRP1 and P-gp expression were associated with well and moderately differentiated histology (*P*<0.0001 and *P*=0.03, respectively) and intestinal type (*P*<0.0001 and *P*=0.009, respectively). High MRP1 expression correlated with lymph node metastasis (*P*=0.037), advanced stage (*P*=0.015), and older age (*P*=0.021). There was no significant association between expression of MRP1 and P-gp and other patient and tumour characteristics (Table 1

**Table 1:**
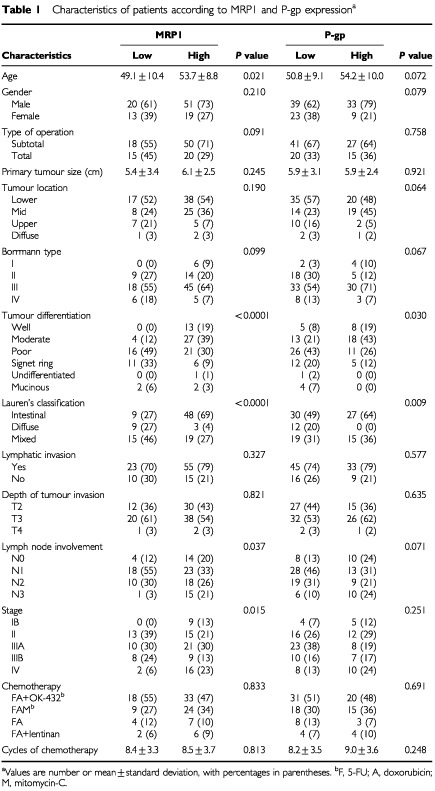
Characteristics of patients according to MRP1 and P-gp expression

). MRP1 and P-gp expressions were significantly associated with each other (*P*<0.0001) (Table 2

**Table 2:**
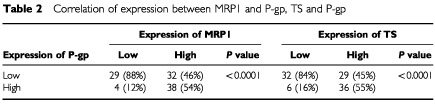
Correlation of expression between MRP1 and P-gp, TS and P-gp

). There was also significant association between P-gp and TS expression (*P*<0.0001) (Table 2). Multidrug resistance-associated protein1 and TS did not correlate with each other (*P*=0.094).

### Association of expression of drug resistance proteins with outcome of patients

The median follow-up duration of the survivors was 68 months (range: 53–80 months) and no patient was lost to follow-up. At the time of analysis, 40 patients had recurrences, and two had second primary cancer (hepatocellular carcinoma and laryngeal cancer, respectively). Forty-seven of the 103 patients have died. Death due to recurrence of gastric cancer occurred in 40 patients. Two patients died of gastrointestinal bleeding and pneumonia, respectively, without evidence of recurrence, one patient died of second primary cancer (hepatocellular carcinoma), and the causes of death were undetermined in four patients.

**Figure 2:**
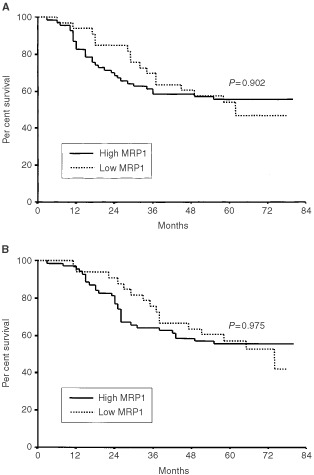
Disease-free survival (**A**) and overall survival (**B**) of gastric cancer patients according to MRP1 expression.

Five-year DFS and OS of total patients were 55.2% and 56.2%, respectively. There were no significant differences in DFS and OS according to the expression of MRP1 (*P*=0.902 and *P*=0.975, respectively), P-gp (*P*=0.987 and *P*=0.955, respectively), and TS (*P*=0.604 and *P*=0.802, respectively) (Figure 2

Figure 3

**Figure 3:**
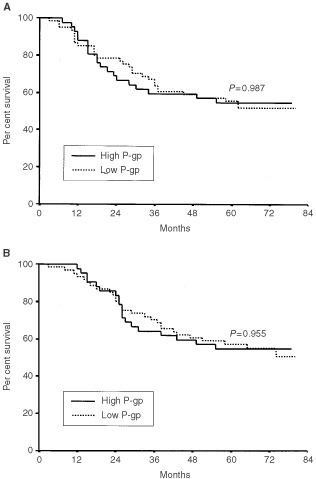
Disease-free survival (**A**) and overall survival (**B**) of gastric cancer patients according to P-gp expression.

Figure 4

**Figure 4:**
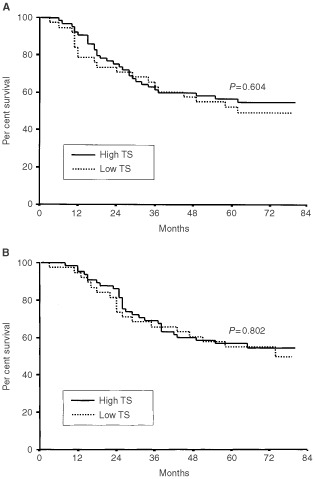
Disease-free survival (**A**) and overall survival (**B**) of gastric cancer patients according to TS expression.

). Concurrent high expression of these proteins (high MRP1 and P-gp, high MRP1 and TS, high P-gp and TS) did not correlate with DFS or OS (Table 3

**Table 3:**
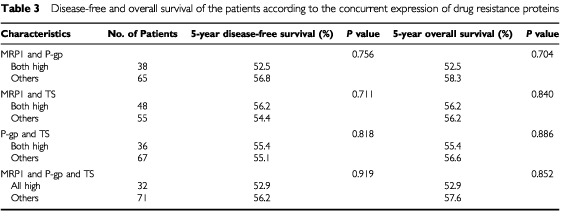
Disease-free and overall survival of the patients according to the concurrent expression of drug resistance proteins

). Even high expression of all three proteins was not associated with poor DFS (*P*=0.919) and OS (*P*=0.852) (Table 3). The prognostic significance of other clinicopathologic characteristics was previously reported (Choi *et al*, 2001). There were no significant differences in DFS and OS according to other characteristics except size (*P*=0.024 and *P*=0.017, respectively) and location (*P*=0.019 and *P*=0.027, respectively) of primary tumour with extended follow-up of patients.

## DISCUSSION

In this study, we evaluated the clinical significance of MRP1, P-gp, and TS known to be associated with resistance of tumours to 5-FU and doxorubicin in locally advanced gastric cancer patients treated with 5-FU and doxorubicin-based adjuvant chemotherapy after curative resection. The association between TS expression and clinicopathologic characteristics was previously reported (Choi *et al*, 2001).

In terms of MRP1 expression in primary tumours, 68% of tumours demonstrated high expression of MRP1. According to other studies, MRP1 expression was reported in 34–55% of patients with gastric cancer (Endo *et al*, 1996a,b; Takebayashi *et al*, 1998; Alexander *et al*, 1999). Multidrug resistance-associated protein1 expression was associated with well and moderately differentiated histology and intestinal type, which was compatible with previous reports (Takebayashi *et al*, 1998; Alexander *et al*, 1999). Furthermore, a significant correlation was found between MRP1 expression and advanced stage. While there was no report showing such relationship in gastric cancer, a study demonstrated a significant association between high expression of MRP1 and advanced stage in breast cancer suggesting the role of MRP1 as a marker of aggressive tumour behaviour (Filipits *et al*, 1996). The mean age of patients with high MRP1 expression was higher than that of patients with low MRP1. This result has relevance to a higher proportion of intestinal type carcinoma, which occurs more often in old patients, in high MRP1 group compared to low MRP1 group (Fuchs and Mayer, 1995). Furthermore, patients with intestinal type tumour showed a higher mean age compared to those with diffuse type in the present study (data not shown).

High expression of P-gp was observed in 41% and also associated with well and moderately differentiated histology and intestinal type as in MRP1. Similar proportion of high expression of P-gp and correlation with well differentiated tumour have been reported in other studies (Mizoguchi *et al*, 1990; Fujii *et al*, 1995; Monden *et al*, 1997; Motoo *et al*, 1998). One interesting finding was a strong association between MRP1 and P-gp expression. While two studies showed no significant correlation between MRP1 and P-gp in gastric cancer, conflicting results were reported in breast cancer (Filipits *et al*, 1996; Beck *et al*, 1998; Dexter *et al*, 1998; Alexander *et al*, 1999; Fan *et al*, 2000; Ferrero *et al*, 2000). The present study suggests that both MRP1 and P-gp are frequently expressed in well and moderately differentiated and intestinal type gastric cancer and closely associated with each other. There is a possibility that MRP1 and MDR1 gene may be coordinately regulated in gastric cancer tissue. There was also a significant correlation between P-gp and TS expression. It is an unexpected finding and further studies would be necessary to find a possible explanation for such a result.

In gastric cancer, the prognostic significance of TS, MRP1, and P-gp expression has been showing conflicting results (Endo *et al*, 1996a; Monden *et al*, 1997; Kuniyasu *et al*, 1998; Takebayashi *et al*; 1998; Suda *et al*, 1999; Choi *et al*, 2001). While TS expression predicted increased risk of recurrence and poor survival in patients treated with adjuvant chemotherapy after surgical resection in two studies, our previous study showed no significant differences in DFS and OS between high TS and low TS group (Kuniyasu *et al*, 1998; Suda *et al*, 1999; Choi *et al*, 2001). There are two studies that investigated the association between MRP1 expression and survival of patients treated with surgery, which failed to demonstrate the prognostic significance of MRP1 expression (Endo *et al*, 1996a; Takebayashi *et al*; 1998). On the other hand, a study suggested that high expression of P-gp was poor prognostic factor in gastric cancer patients who underwent surgical resection followed by adjuvant chemotherapy (Monden *et al*, 1997).

There were no significant differences in DFS and OS of gastric cancer patients according to the expression of MRP1, P-gp, and TS in the present study. We also investigated the prognostic significance of concurrent expression of these three drug resistance proteins. However, concurrent expression of drug resistance proteins did not predict increased recurrence or poor survival. Even high expression of all three proteins was not associated with poor prognosis. These results are a somewhat unexpected find considering the fact that we simultaneously investigated the clinical relevance of well-established drug resistance proteins, which are known to be involved in resistance of tumours to 5-FU and doxorubicin. The lack of prognostic implication of TS, MRP1, and P-gp expression in gastric cancer patients who underwent adjuvant chemotherapy could be explained as follows. The expression of these drug resistance proteins in primary tumours of gastric cancer patients may not reflect the drug resistance of microresidual or micrometastatic cancer cells which cause the recurrence (Choi *et al*, 2001). There is also a possibility that the gastric cancer patients with high expression of drug resistance proteins might experience greater survival benefit from adjuvant chemotherapy compared to patients with low expression as shown in other studies that investigated the expression of TS in rectal cancer and breast cancer (Johnston *et al*, 1994; Pestalozzi *et al*, 1997). However, a larger study including patients treated with surgical resection alone would be essential to clearly define the prognostic implication of these drug resistance proteins in adjuvant chemotherapy of gastric cancer.

To our knowledge, the present study is the first report that investigated the expression of TS, MRP1, and P-gp simultaneously in gastric cancer patients who underwent 5-FU and doxorubicin-based adjuvant chemotherapy. In other studies that investigated the clinical relevance of MRP1 or P-gp, the patients usually received fluoropyrimidine with or without mitomycin-C, in which neither MRP1 nor P-gp is associated with drug resistance (Goldstein *et al*, 1989; Cole *et al*, 1992; Gottesman and Pastan, 1993; Endo *et al*, 1996a; Monden *et al*, 1997; Hipfner *et al*, 1999). Our results suggest the possibility that the expression of drug resistance-related proteins has little clinical significance in gastric cancer patients who received adjuvant chemotherapy. Similarly, a study that investigated the expression of MRP1 and MDR1 mRNA simultaneously in node-positive breast cancer patients who received anthracycline-based adjuvant chemotherapy, expression of MRP1 and MDR1 had no significant influence on survival (Ferrero *et al*, 2000).

In conclusion, high expression of MRP1, P-gp, and TS including concurrent expression did not predict poor DFS and OS in patients with locally advanced gastric cancer treated with 5-FU and doxorubicin-based chemotherapy after curative resection. A larger study including patients treated with surgical resection alone would be necessary.
